# Relationships Between Copper-Related Proteomes and Lifestyles in β Proteobacteria

**DOI:** 10.3389/fmicb.2019.02217

**Published:** 2019-09-24

**Authors:** Rudy Antoine, Alex Rivera-Millot, Gauthier Roy, Françoise Jacob-Dubuisson

**Affiliations:** Université de Lille, CNRS, INSERM, CHU Lille, Institut Pasteur de Lille, U1019 – UMR 8204 – Center for Infection and Immunity of Lille, Lille, France

**Keywords:** copper homeostasis, metalloproteome, *in silico* analyses, β proteobacteria, lifestyles

## Abstract

Copper is an essential transition metal whose redox properties are used for a variety of enzymatic oxido-reductions and in electron transfer chains. It is also toxic to living beings, and therefore its cellular concentration must be strictly controlled. We have performed *in silico* analyses of the predicted proteomes of more than one hundred species of β proteobacteria to characterize their copper-related proteomes, including cuproproteins, i.e., proteins with active-site copper ions, copper chaperones, and copper-homeostasis systems. Copper-related proteomes represent between 0 and 1.48% of the total proteomes of β proteobacteria. The numbers of cuproproteins are globally proportional to the proteome sizes in all phylogenetic groups and strongly linked to aerobic respiration. In contrast, environmental bacteria have considerably larger proportions of copper-homeostasis systems than the other groups of bacteria, irrespective of their proteome sizes. Evolution toward commensalism, obligate, host-restricted pathogenesis or symbiosis is globally reflected in the loss of copper-homeostasis systems. In endosymbionts, defense systems and copper chaperones have disappeared, whereas residual cuproenzymes are electron transfer proteins for aerobic respiration. Lifestyle is thus a major determinant of the size and composition of the copper-related proteome, and it is particularly reflected in systems involved in copper homeostasis. Analyses of the copper-related proteomes of a number of species belonging to the *Burkholderia*, *Bordetella*, and *Neisseria* genera indicates that commensals are in the process of shedding their copper-homeostasis systems and chaperones to greater extents yet than pathogens.

## Introduction

β proteobacteria form a large phylogenetic group mainly composed of environmental species and a few important pathogens, notably of the *Burkholderia*, *Bordetella*, and *Neisseria* genera. β proteobacteria also comprise a few known phytopathogens, commensals, endophytes, symbionts, and endosymbionts. Thus, members of this phylogenetic group represent a broad range of lifestyles, though information is scarce for most identified species.

Copper is a transition metal whose redox properties are widely used notably in electron transfer chains for respiration and photosynthesis, and in enzymes involved in oxido-reduction and hydrolytic reactions. Thus, bacteria need to acquire copper ions from the milieu (Stewart et al., 2019). However, Cu(I), which can cross the cytoplasmic membrane, is toxic at high concentrations (Fu et al., 2014; Djoko et al., 2015). Toxicity is thought to be caused by the reactive hydroxyl radical generated in Fenton and Haber-Weiss type reactions, and by copper displacing iron from its sites in metallo-proteins (Solioz, 2018). In particular, the biogenesis of 4Fe-4S centers is vulnerable to copper (Macomber and Imlay, 2009), and copper can also displace iron from assembled 4Fe-4S clusters (Azzouzi et al., 2013; Djoko and McEwan, 2013). As a consequence of this toxicity, bacteria have developed ways to strictly control intracellular copper concentrations (Nies, 2003; Ladomersky and Petris, 2015). A number of systems that ensure copper homeostasis, including extrusion of copper from the cytoplasm or from the periplasm and oxidation of Cu(I) into less toxic Cu(II), have been described in a few model bacteria including *Escherichia coli*, *Salmonella typhimurium*, *Pseudomonas aeruginosa*, *Enterococcus hirae*, and *Mycobacterium tuberculosis* (Wolschendorf et al., 2011; Fu et al., 2014). In contrast, little in known for most other bacteria (Solioz, 2018).

Expression of copper-related proteins is controlled by specific two-component signal transduction systems and transcriptional regulators. Typically, transcription of genes or operons involved in the export of copper or its detoxification is activated when excess copper is detected in the periplasm using two-component systems such as CusRS or CopRS, or in the cytoplasm using regulators of the MerR, ArsR, or CsoR families (Ma et al., 2009; Capdevila et al., 2017; Chandrangsu et al., 2017). Acquisition systems in case of copper starvation are less well-known. Few specific import systems of copper have been described, and they are mainly dedicated to the assembly of respiration and denitrification complexes (Ekici et al., 2012; Khalfaoui-Hassani et al., 2018).

β proteobacteria include *Cupriavidus metallidurans*, aptly named from its capacity to survive in environments heavily contaminated with transition metals, as it was first isolated from the sludge of a decantation tank in a zinc factory (Mergeay et al., 1985). This organism is extremely well equipped to deal with excessive concentrations of those elements including copper, either by efflux, complexation or reducing precipitation (Von Rozycki and Nies, 2009; Grosse et al., 2016; Herzberg et al., 2016). Rather far from this type of niche is the obligate, host-restricted pathogen *Bordetella pertussis*, the whooping cough agent (Melvin et al., 2014; Linz et al., 2019). *B. pertussis* lives in the respiratory mucosa of humans, mainly as an extracellular pathogen. It has no known environmental reservoir and is believed to be transmitted directly between humans. We have initiated the study of copper homeostasis in *B. pertussis* and discovered that it has considerably streamlined its defense against copper relative to model pathogenic bacteria. *Bordetella bronchiseptica*, a close relative of *B. pertussis* with a larger genome and a more promiscuous lifestyle, that can survive in the environment in addition to infecting mammals (Taylor-Mulneix et al., 2017), has more copper-regulated defense systems against excess of this metal than *B. pertussis* (our unpublished observations). This finding prompted us to analyze the predicted copper-related proteomes of a large range of β proteobacteria to investigate more broadly the links between their lifestyles and the homeostasis of this metal.

## Materials and Methods

### Retrieval of β Proteobacterial Proteomes

All β proteobacterial species with genomic sequences in the NCBI database (release Nov 2018) were collected, resulting in 465 distinct species. Among them, those for which the genomes are completely sequenced were selected. A single bacterial species was selected for most genera, based on the numbers of publications available in the Pubmed database on each species. However, we selected one representative isolate of all the species of the three β proteobacterial genera – *Bordetella*, *Burkholderia* and *Neisseria-* that include important pathogens. The RefSeq genome files of the selected species were retrieved whenever available, and assembled genome files were used in the other cases. We generated the predicted proteomes of the selected species by translating all their annotated open reading frames. The proteins thus obtained were analyzed in the CLC main package to predict protein domains according to the Pfam nomenclature.

### *In silico* Searches for Cu-Related Domains

An exhaustive search was conducted to identify all types of known copper-related protein domains that can be found in β proteobacteria. Instead of using a Blast approach to retrieve putative members of known copper-related protein families, we used family signatures as found in Pfam. Firstly, all proteins whose three-dimensional structures contain a copper ion were retrieved from the metal-specific MetalPDB database (Putignano et al., 2018) as described previously (Sharma et al., 2018). The 1397 distinct 3-dimensional structures of proteins with bound Cu were found to correspond to 4391 protein sequences, as some entries include several polypeptide chains. Pfam predictions were performed for all sequences, and 233 distinct Pfam domains were identified in that set. Among those, we determined which domains provide amino acyl residues that coordinate copper. The binding sites of Cu are mainly formed by the side chains of His, Cys, and Met residues (Rubino et al., 2011), as well as Asp and Glu according to MetalPDB. A metal binding site was considered plausible if all coordinating residues belong to a single predicted Pfam domain, yielding 27 potential Cu-binding domains. Mismetallated domains and eukaryote-specific domains were discarded.

Secondly, we used the BACMET database to identify additional Cu-related Pfam domains absent from MetalDB as described (Li et al., 2019). In BACMET, 97 proteins are reported to be involved in copper resistance in eubacteria, but this list is highly redundant. This additional search identified two new domains, CopD and CopB. BACMET also led to the identification of domains involved in metal sensing, in particular histidine sensor-kinases of two-component systems and cytoplasmic transcriptional regulators of the MerR family. However, we chose to disregard metal-sensing domains of regulation systems in our analyses, because of the difficulty to determine their metal specificity based on sequence alone (Ibanez et al., 2015). Functional studies and three-dimensional structures are generally required to establish selectivity (Pennella et al., 2003; Ma et al., 2009).

Thirdly, we searched the Pfam database by text mining for copper-related domains that might have been missed in the first two approaches. A few additional domains were found, namely CutC, NosL, Cu-oxidase_4, and NnrS.

After having identified all known copper-related domains, we searched for their presence in each of our predicted β proteobacterial proteomes using hmmsearch^1^. For most domains, there is no ambiguity regarding their specificity for copper. However, for others further analyses were necessary. To identify metal-specific exporters, we retrieved all the putative proteins identified as P1-type ATPases (domain E1-E2_ATPases) and RND (domain ACR-tran) transporters according to Pfam in our set of proteins. We performed BlastP analyses against the 17545 proteins of the TCDB database^2^ (link: TCDB FastA Sequences), which provides subcategories of transporters according to their substrates. Only hits with E values of 0.0 were selected, namely the copper resistance ATPases (TCDB category 3.A.3.5) and the metal-specific RND HME transporters (heavy metal efflux; TCDB 2.A.6.1). Note that ATPases providing copper to respiratory complexes (Gonzalez-Guerrero et al., 2010; Hassani et al., 2010) are part of a distinct TCDB category (3.A.3.27) that we did not consider in our analysis.

Multicopper oxidases (MCO) harbor at least two copper-containing domains that can also be found in other types of proteins of various functions. X-ray structures of *bona fide* MCOs were analyzed to determine their specific domain organizations, which are Cu-oxidase_3/Cu-oxidase/Cu-oxidase_2; Cu-oxidase_3/Cu-oxidase_2; and Cu-oxidase_3//Cu-oxidase_2. The same approach was used for copper-containing nitrite reductases, yielding the following domain organizations:

Cu-oxidase_3/Cu-oxidase; Cu-oxidase_3//Cytochrome_CB B3; Copper-bind/Cu-oxidase_3; Copper-bind/Cu-oxidase_3/Cu-oxidase_2. For nitrous oxide reductases (N_2_OR), the nos_propeller domain was used as the defining signature according to MetalPDB.

Three complexes of aerobic respiration, cytochrome bo ubiquinol oxidases, cytochrome C oxidases aa3, and cytochrome C oxidases cbb3, use copper and contain a COX1 domain. Cytochrome C oxidases cbb3 can be defined by the presence of a FixO Pfam domain in one of their components. TIGRFAM^3^ was used to determine the identities of the other two complexes. Cytochrome bo ubiquinol oxidases have a component with a CyoB domain signature (TIGR02843). For cytochrome oxidase aa3 identification, the TIGRFAM signature QoxB (TIGR02882) was used. As some nitric oxide reductases also harbor this domain, we used the KEGG oxidative phosphorylation and nitrogen metabolism reference pathways to assign unambiguously the cytochrome C oxidases aa3 in our set of proteomes.

For Copper Storage Proteins (CSPs) (Dennison et al., 2018), the only available signature is a domain of unknown function (DUF326; PF02860), and therefore we created a new, unique CSP signature. A DUF326 domain is found in 18 proteins of our set that are annotated in GenBank RefSeq files as “four-helix bundle copper-binding protein.” Using those keywords, 22 additional proteins were identified. Sequence alignments of the 40 proteins with ClustalW was used to create a new CSP profile, called CSP.hmm (Supplementary File S1), using hmmbuild^1^. Searching for this profile in our proteomes retrieved seven additional proteins. As CSPs can be periplasmic or cytoplasmic, they were distinguished based on the prediction of a Tat signal peptide using the Tatfind server^4^. Eight proteins belong to the extracytoplasmic group (Csp1/2_Ecsp), and the other 39 are cytoplasmic (Csp3_Ccsp).

### Hierarchical Clustering Analyses

Hierarchical clustering was performed using the Cluster 3.0 software^5^ to group bacteria based on the occurrence and abundance of the various types of copper-related proteins. We used medians of copy numbers of each type of proteins in each species and dispersion of the values around the medians for hierarchical clustering, with the Correlation (uncentered) similarity metric parameter. All other parameters were kept at their default values. Hierarchical clustering was also used to identify co-occurrences of the various proteins in our bacterial set without centering around medians. Note that those analyses were performed on 86 species, as only one species was selected for the *Burkholderia*, *Bordetella*, and *Neisseria* genera to avoid their overrepresentation in our bacterial set. The dendrograms were exported to Figtree for visualization^6^.

### Phylogenetic Analyses

A phylogenetic tree was built based on 16S RNA sequences using one species for each genus (86 sequences). They were aligned with cmalign of the Infernal package^7^ using the bacterial small subunit ribosomal RNA profile (SSU_rRNA_bacteria, RF0077). The MEGA X software^8^ (Kumar et al., 2018) was used to build a Neighbor-joining-tree using the Maximum likelihood algorithm with the Tamura-Nei nucleotide substitution model (Tamura and Nei, 1993) and 1000 replicates using the Bootstrap method (Felstenstein, 1985). The phylogenetic tree was visualized with FigTree.

## Results

### Diversity of β Proteobacteria

After collecting all β proteobacterial species whose genomes are completely sequenced, we selected one species for each genus in order to obtain as wide a range of lineages as possible while keeping the analyses to a manageable level. For the *Bordetella*, *Neisseria*, and *Burkholderia* genera, we selected one isolate as representative of each species. Altogether, this yielded 119 distinct species of β proteobacteria, with the largest possible variety of genera and lifestyles, including a few unclassified species (Supplementary Table S1). As no completely sequenced genomes from the Ferritrophicales, Ferrovales, and Procabacteriales orders were available at the time of our analyses, no representatives of those phylogenetic groups were included.

Many species in our set are described to live in natural milieus such as water and soils and will be called hereafter environmental bacteria, although limited information is available in many cases. The environmental species reported to frequently cause infections in specific conditions were placed in a category called environmental/opportunists. *Bona fide* pathogens were sorted according to their types of hosts, yielding animal pathogens and phytopathogens. The single species of fungus pathogen was included in the group of environmental bacteria. We also identified a few endophytes, symbionts, endosymbionts, and commensal species. Commensals responsible for opportunistic infections were placed in a separate category.

**FIGURE 1:**
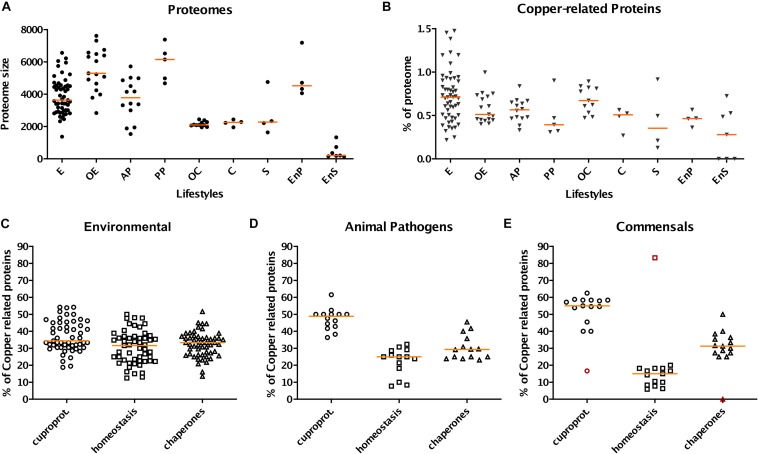
Profiles of copper-related proteomes as a function of the lifestyles of β proteobacteria. The bacteria were grouped according to their lifestyles, and various data were extracted from their predicted proteomes. E, OE, AP, PP, OC, C, S, EnP, and EnS represent environmental, opportunistic/environmental, animal pathogen, phytopathogen, opportunistic/commensal, commensal, symbiotic, endophytic, and endosymbiotic bacteria, respectively. **(A)** Sizes (in numbers of proteins) of the total proteomes as a function of the lifestyles of β proteobacteria. **(B)** Proportions (in%) of the copper-related proteomes relative to the entire proteomes. The copper-related proteomes include all predicted proteins of the three categories described in the text (cuproproteins, copper-homeostasis proteins, and copper chaperones). **(C–E)** Proportions of cuproproteins, copper-homeostasis proteins, and copper chaperones relative to the copper-related proteomes in selected bacterial groups, namely environmental bacteria **(C)**, animal pathogens **(D)**, and commensals **(E)**. Note that in panel E both commensals and commensals/opportunists were included in order to increase the number of species in that group. The red symbols in panel E correspond to *O. formigenes*. In each panel, the different species are each represented by one symbol. The orange horizontal lines represent the medians of the values for the various groups.

We generated the complete predicted proteomes of all selected isolates and determined the Pfam domain(s) present in each protein. The largest proteome sizes in the various categories of bacteria are those of opportunists, environmental/opportunists and pathogens, although there are considerable variations within each of those groups (Figure 1A). Endosymbionts are at the other end of the spectrum, as expected. Strikingly, the proteome size of *Verminephrobacter eisiniae*, a heritable extracellular earthworm symbiont, is similar to those of environmental β Proteobacteria (Pinel et al., 2008; Lund et al., 2014).

### Identification of Copper-Related Proteins in the Selected Set of β Proteobacteria

We inventoried all predicted proteins that harbor Cu-related domain(s). As several of those domains are frequently associated with one another, some proteins or protein complexes in our set include more than one Cu-related domain (Table 1). Copper-related proteins were sorted in several categories. The first encompasses cuproproteins, i.e., proteins that harbor copper ion(s) necessary for their activity. Fourteen distinct families of cuproproteins are present in β proteobacteria. The second group corresponds to proteins mediating copper homeostasis, in particular defense against copper. Resistance to excess copper mainly consists in transporting it from the cytoplasm to the periplasm via P_1__*B*_-type copper ATPases (Migocka, 2015) and from the periplasm to the external milieu via heavy metal exporters (HME) of the resistance/nodulation/division (RND) superfamily of transporters (Kim et al., 2011). An additional line of defense is to detoxify Cu(I) by oxidation to Cu(II) using multi-copper oxidases (MCOs) (Singh et al., 2004; Kaur et al., 2019). Note that in addition to mediating Cu homeostasis, MCOs are *bona fide* cuproproteins.

**TABLE 1:** Copper-related proteins identified in β Proteobacteria.

	**Name**	**Other names**	**Pfam name**	**Pfam IDs**
Cuproproteins	Nitrite reductase		Cu-oxidase,Cu-oxidase_2,Cu-oxidase_3^∗^	PF00394,PF07731,PF07732
	Nitrous oxide Reductase		nos_propeller	PF18764
	Nnrs		Nnrs	PF05940
	Copper-bind	Plastocyanin/azurin	Copper-bind	PF00127
	Cu_amine_oxid		Cu_amine_oxid	PF01179
	Cu-oxidase_4	laccase	Cu-oxidase_4	PF02578
	Cupredoxin_1		Cupredoxin_1	PF13473
	DUF386		DUF386	PF04074
	Monooxygenase_B		Monooxygenase_B	PF04744
	Sod_Cu	Cu Superoxide Dismutase	Sod_Cu	PF00080
	Tyrosinase		Tyrosinase	PF00264
	Cyto_bo		COX1,COX2^∗^	PF00115,PF00116
	Cyto_C_aa3		COX1,COX2^∗^	PF00115,PF00116
	Cyto_C_cbb3		COX1,FixO^∗^	PF00115,PF02433

Homeostasis	MCOs		Cu-oxidase,Cu-oxidase_2,Cu-oxidase_3^∗^	PF00394,PF07731,PF07732
	Cu ATPases		E1-E2_ATPases^∗^	PF00122
	RNDs HME	Heavy Metal Efflux RND Exporters	ACR tran^∗^	PF00873
	CopB		CopB	PF05275
	CopC		CopC	PF04234
	CopD		CopD	PF05425
	CopK		CopK	PF11525
	CutA1		CutA1	PF03091
	CutC		CutC	PF03932

Chaperones	Csp3_Ccsp	Cytoplasmic Copper Storage Protein	this work^∗^	
	CusF_EC		CusF_EC	PF11604
	HMA	CopZ	HMA	PF00403
	PCuAC		PCuAC	PF04314
	SCO1-SenC		SCO1-SenC	PF02630
	Csp1/2_Ecsp	Extracellular Copper Storage Protein	this work^∗^	
	CtaG_COX11		CtaG_COX11	PF04442
	NosL		NosL	PF05573

*The proteins were sorted by functional categories: cuproproteins, copper-homeostasis proteins, and copper chaperones. The Pfam names and identity numbers of the domains that interact with copper in each protein are provided when available. Note that multicopper oxidases are both cuproproteins and copper homeostasis proteins, and Csp3_Ccsp were placed in both homeostasis proteins and copper chaperones. Asterisks indicate proteins whose definition required additional *in silico* analyses (see section “Materials and Methods”).*

Regarding the other facet of copper homeostasis, i.e., import of copper as a micronutrient, few systems have been described. Reports that specific P_1__*B*_-type copper ATPases might import rather than expel copper have thus far not been convincingly substantiated (Solioz, 2018). Specific major facilitator superfamily (MFS) importers take up copper across the cytoplasmic membrane, notably for cytochrome C oxidase assembly (Ekici et al., 2012; Khalfaoui-Hassani et al., 2018). TonB-dependent receptors (TBDR) that transport copper across the outer membrane have also been reported, notably in *Pseudomonas* (Yoneyama and Nakae, 1996; Wunsch et al., 2003). However, as the MFS and TBDR families include many paralogs but no clear signatures specify copper transporters, we chose to not include those two classes of transporters in this analysis. Other proteins potentially involved in copper homeostasis include the periplasmic and inner membrane proteins CopC and CopD, respectively, reported to form a system that sequesters copper in the periplasm (Cooksey, 1994), and other proteins of ill-defined functions, the outer membrane protein CopB (Lawaree et al., 2016) and the periplasmic protein CopK (Monchy et al., 2006).

The third category includes copper-specific chaperones (Robinson and Winge, 2010; Capdevila et al., 2017), most of which play more than one role. Cu chaperones transfer copper for cuproprotein assembly or participate in copper extrusion by handing copper over to exporters. They also buffer copper, which may contribute to limiting its toxicity (Corbett et al., 2011; Osman et al., 2013; Vita et al., 2016; Solioz, 2018; Novoa-Aponte et al., 2019; Utz et al., 2019). We chose to place Csp3_Ccsp (cytosolic copper storage proteins) both in the homeostasis and copper chaperone categories, based on its putative functions (Dennison et al., 2018).

A first observation is that the proportion of copper-related proteins in a given species is not necessarily related to its proteome size (Figures 1A,B). Among those, cuproproteins account for 40% of copper-related proteins, homeostasis proteins for 28% and copper chaperones for 32%, using medians. However, those proportions vary depending on the lifestyles. Thus, pathogens, commensals and symbionts have greater proportions of cuproproteins and chaperones and lower proportions of homeostasis systems than environmental bacteria (Figures 1C–E, Supplementary Figure S1 and Supplementary Table S2). Endophytes and symbionts have few copper-related proteins, which are mostly cuproproteins, and hardly any homeostasis systems (Supplementary Figure S1 and Supplementary Table S2). For phytopathogens, the dispersion of the values is too large and the size of the samples is too small to identify trends.

**FIGURE 2:**
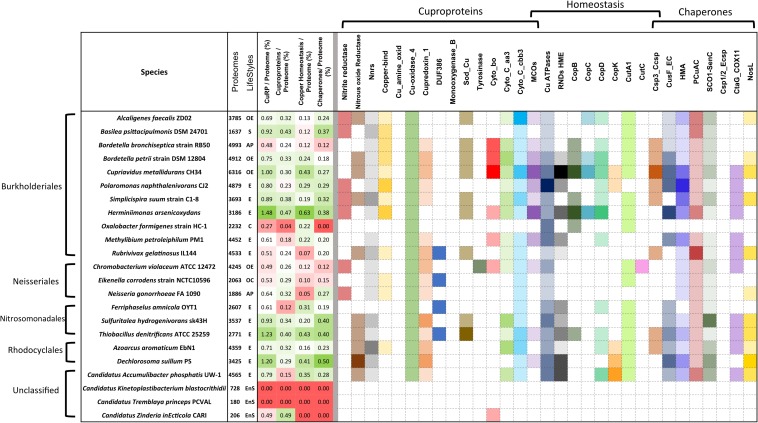
Copper-related proteomes in representative species of β proteobacteria. A limited number of species was selected that represent various orders of β proteobacteria (Burkholderiales, Neisseriales, Rhodocyclales, Nitrosomonadales, and unclassified) and various lifestyles. The full set of data describing the 119 species is provided in Supplementary Figure S1. The sizes of the proteomes (numbers of proteins) and the lifestyles are given in the second vertical panel, with the same abbreviations as in the legend to Figure 1. In the third vertical panel are shown the proportions (in%) of the copper-related proteomes (CuRP) relative to the complete proteomes for each species, and the proportions (in%) of the proteins in the three functional categories (cuproproteins, copper-homeostasis proteins, and copper chaperones) relative to the complete proteomes. In that panel, the white, green, and red background colors represent average values, above-average values, and below-average values, respectively, relative to the averages for the 119 species. The intensities of the colors are proportional to the distances to the average values. The abundances of each type of proteins in each bacterial species are represented in the last panel. Note that MCOs are both cuproproteins and copper-homeostasis proteins, and Csp3_Ccsps (cytoplasmic copper storage proteins) are both copper-homeostasis proteins and copper chaperones. The intensities of the colors increase with the number of paralogs in each bacterial species, with white indicating the absence of the protein. The absolute numbers of proteins of each type in each bacterial species can be found in Supplementary Table S2.

In environmental bacteria, by far our largest sample, the situation is rather contrasted, likely depending on their respective niches. Using medians, copper-related proteins make up 0.71% of their proteomes, of which 34, 33, and 33% are cuproproteins, copper-homeostasis proteins, and copper chaperones, respectively (Figures 1B,C). Nitrosomonadales have particularly high proportions of proteins of all three categories relative to the sizes of their proteomes (Supplementary Table S2). Some Burkholderiales including *C. metallidurans* are well equipped to deal with excess copper. The champion is *Herminiimonas arsenicoxydans*, with 1.48% of its medium-size proteome made of copper-related proteins (Figure 2).

### Analysis by Types of Proteins

Use of copper in β proteobacteria is largely linked to aerobic respiration, as the most represented cuproproteins are cytochrome C oxidases aa3 and cbb3 (Figure 2 and Supplementary Table S2 and Supplementary Figure S1). Very few species in our set lack both types of cytochrome C oxidases, including one uncharacterized *Bordetella* species. Cytochromes bo ubiquinol oxidase complexes are present in half of our bacterial set. The only bacteria totally devoid of genes for aerobic respiration are *Oxalobacter formigenes*, a commensal of the human digestive tract, and two endosymbiont candidates with minute proteomes, *Tremblaya princeps* and *Vidania fulgoroideae*.

Neisseriales have few cytochrome C oxidases or ubiquinol oxidases, but they possess complexes for respiration on oxidized nitrogen species two of which, nitrite reductase (Mellies et al., 1997) and N_2_OR, are cuproproteins. Genes for those proteins are also found in some environmental bacteria, including ammonium-oxidizing bacteria (Casciotti and Ward, 2001), some Rhodocyclacles and some Burkholderiales (Figure 2 and Supplementary Figure S1). NnrS is a cuproprotein linked to nitrosative stress (Patra et al., 2019). It is present in most β proteobacteria, at several copies in Rhodocyclales, Nitrosomonadales, Neisseriales, and some Burkholderiales.

Small copper-containing plastocyanin-like electron transfer proteins and cupredoxins are also broadly present in β Proteobacteria, with genes found at 1–3 copies in more than half of the genomes. Largest numbers are in environmental bacteria (Supplementary Table S2).

Periplasmic MCOs are both cuproproteins and proteins involved in copper homeostasis, using O_2_ to mediate oxidation of Cu(I) to Cu(II) as a means to reduce its toxicity. The numbers of MCO-coding genes vary from 0 in symbionts, some animal pathogens and anaerobes, to four or five in *C. metallidurans*, *H. arsenicoxidans*, *Ralstonia solanacearum*, and *Nitrosomonas europaea* (Supplementary Table S2).

Cu, Zn superoxide dismutases (SodC) protect the cell from superoxide stress, either exogenous or endogenous (Battistoni, 2003; Broxton and Culotta, 2016; Larosa and Remacle, 2018). *sodC* genes are found in half of the species, with two copies in a few environmental species. Most endosymbionts and a majority of Neisseriales are devoid of *sodC*, consistent with micro-aerophilic environments (Figure 2 and Supplementary Figure S1).

A single gene copy for laccase (multicopper polyphenol oxidase) is found in most β proteobacteria. Finally, other categories of cuproproteins are present in more limited numbers of species, most likely for specific metabolisms (Figure 2 and Supplementary Figure S1).

### Cu Homeostasis in β Proteobacteria

Resistance to copper is mediated by several mechanisms involving Cu-specific ATPases, RND transporters, MCOs, in some cases coupled with export across the outer membrane (Lawaree et al., 2016), and sequestration by copper-binding proteins, including copper chaperones, in the two compartments. As chaperones also participate in Cu traffic within cells, they are considered separately.

**FIGURE 3:**
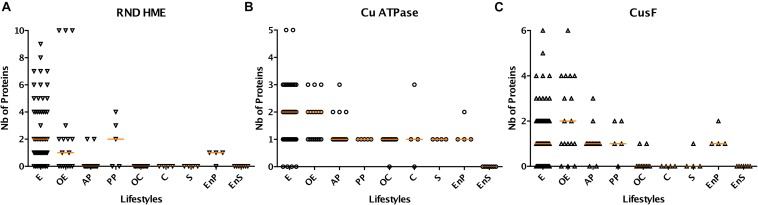
Abundances of selected classes of copper-related proteins (RND HME in **A**, Cu ATPase in **B** and CusF in **C**) in β proteobacteria as a function of their lifestyles. Absolute numbers are shown here, and the horizontal orange lines represent the medians of the values for each group. E, environmental bacteria; OE, opportunistic/environmental bacteria; C, commensal bacteria; OC, opportunistic/commensal bacteria; AP, animal pathogens; PP, phytopathogens; EnP, endophytes; S, symbionts; EnS, endosymbionts. The orange horizontal lines represent the medians of the values for the various groups.

Genes for copper-specific P_1__*B*_-type ATPases are found in 106 species from our set, making those systems the most widespread mechanisms of defense against copper in β proteobacteria. The largest numbers, up to 5, are found in specific environmental bacteria (Figure 3B). Genes for RND-mediated export by heavy metal exporters (HME) are present in 62 species. Up to 10 RND HME-coding genes are present in some environmental bacteria, irrespective of the size of their proteome, a strong indication that those bacteria are exposed to transition metals in their environment (Figure 3A). It is, however, difficult to determine which of those might be involved in copper efflux. In contrast, other environmental bacteria, symbionts, and animal pathogens do not possess such genes at all, indicating that this defense feature is strongly correlated with lifestyle. For instance, Neisseriales and most animal pathogens have no HME genes but have at least one Cu-ATPase gene (Figure 2 and Supplementary Figure S1). As the two types of systems expel copper from different cellular compartments, its removal from the cytoplasm might be sufficient for bacteria that have no environmental phase in their life cycles.

Among other proteins reported to mediate Cu homeostasis, several copies of CopC and CopD are found in bacteria that live in metal-rich environments and in some *Burkholderiae*, whereas symbionts and most animal and phytopathogens are devoid of them (Figure 2). This suggests a role for defense against copper, in line with early reports (Silver and Ji, 1994). Other poorly characterized proteins, including CopB, CopK, CutA1, and CutC are absent from the genomes of most β Proteobacteria or present in low copy numbers, indicating that they likely represent specific adaptations of a limited number of genera.

### Cu Metallochaperones

The isolated HMA domain, called CopZ in model bacteria, has several roles: it sequesters Cu in the cytoplasm and also hands Cu(I) to Cu-ATPases, either as a means of defense or to supply copper for cytochrome C oxidase assembly (Corbett et al., 2011; Utz et al., 2019). Among the single-domain HMA proteins in our set, greatest numbers are found in environmental bacteria including *C. metallidurans*, *H. arsenicoxydans*, and *Polaromonas naphtalenivorans*. Very few β proteobacteria do not have any, supporting its role for copper homeostasis in that phylogenetic group.

CusF is a periplasmic Cu-binding protein that transfers copper to the RND HME transporter CusABC for extrusion of Cu to the external milieu in model bacteria. Largest numbers of *cusF* genes are found in *B. multivorans*, *Alicycliphilus denitrificans*, *H. arsenicoxydans*, *Dechloromonas suillum*, *Polaromonas naphtalenivorans*, i.e., opportunists or environmental bacteria that possess multiple RND HME systems (Figure 3C). However, *cusF* is present in a number of species devoid of RND HMEs, in particular in animal pathogens including all pathogenic *Bordetellae* (Figure 2 and Supplementary Figure S1). It must thus fulfill another role in the absence of RND HME systems.

The PCu_*A*_C and ScoI-SenC chaperones are involved in the assembly of respiration or photosynthetic complexes or of nitrite reductase (Jen et al., 2015), and they have also been reported to participate in copper homeostasis (Trasnea et al., 2016). They are found in most β proteobacterial proteomes, except for *O. formigenes*, *Candidatus Symbiobacter mobilis*, endosymbionts and others that have few or no genes for aerobic respiration (Supplementary Figure S1). NosL is a Cu chaperone involved in the assembly of N_2_OR (Zumft, 2005), and accordingly it is mostly found in species with that enzyme. Finally, genes for copper storage proteins (Dennison et al., 2018) Csp3_Ccsp (cytoplasmic Cu storage) are present in a number of environmental bacteria as well as in several *Bordetellae*. As for Csp1/2_Ecsp (periplasmic Cu storage) proteins, they are only found in a few species, including several Neisseriales (Supplementary Figure S1).

### Co-occurring Copper-Related Proteins in β Proteobacteria

**FIGURE 4:**
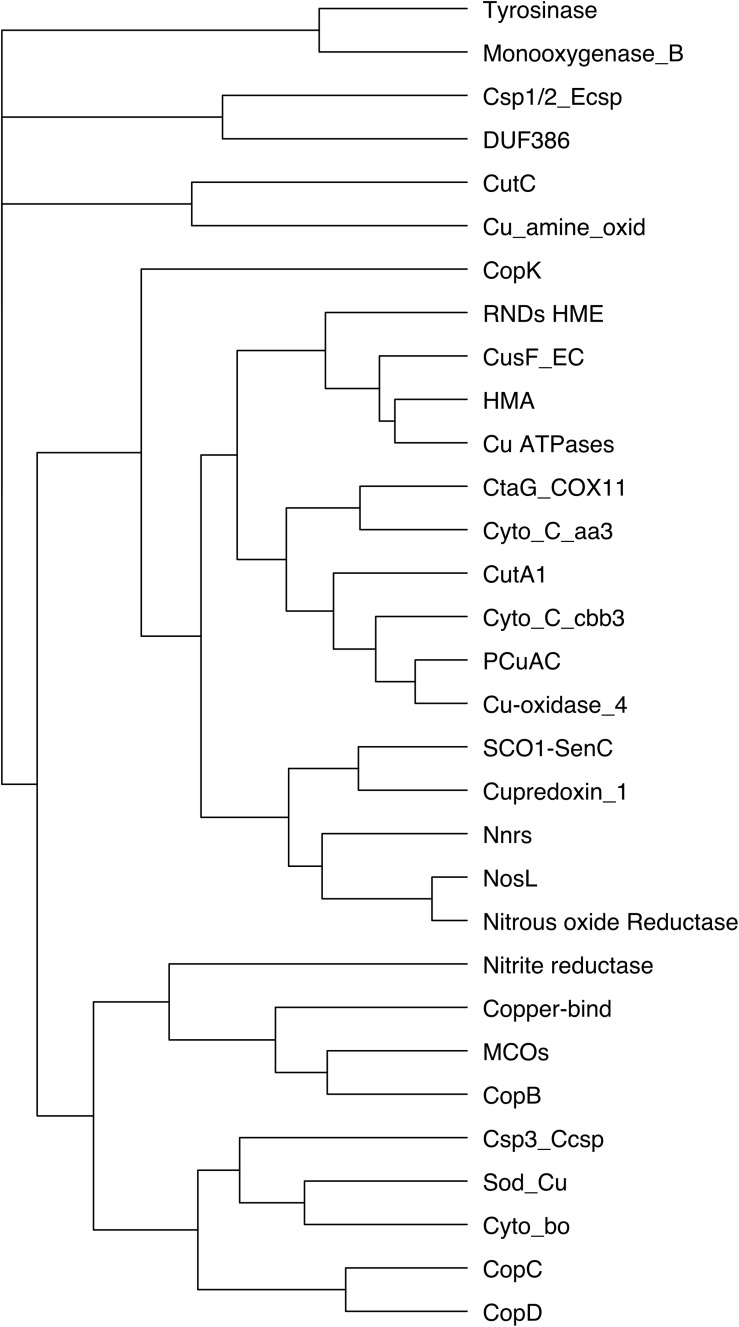
Clustering of copper-related proteins in β proteobacteria. Hierarchical clustering was performed for the 31 protein types and 86 species (including one species of each of the *Neisseria*, *Burkholderia*, and *Bordetella* genera) to identify co-occurrences of proteins in β proteobacteria. FigTree was used to visualize the results. The number of nodes between two proteins is negatively correlated with their co-occurrence.

Hierarchical clustering was performed on 86 species with a single representative of each bacterial genus, including *Burkholderia cepacia*, *B. bronchiseptica*, and *N. gonorrheae.* Most of the co-occurrences of copper-related proteins in that bacterial set revealed by those analyses were expected based on the functions of the respective proteins (Figure 4). Thus, RND HME exporters and CusF, which form export systems across the outer membrane, are often found together. Similarly, isolated HMA domains have been described to transfer copper to Cu-ATPases for export from the cytoplasm, and accordingly, the two proteins cluster in our set. We also observed co-occurrence of RND HME and Cu-ATPases. This is in good agreement with the report that the two systems can synergize for the defense against copper (Padilla-Benavides et al., 2014). The chaperone NosL cluster with N_2_OR, as expected from its role for N_2_OR assembly. Other associations revealed by our analyses include MCO with the OMP CopB, cupredoxin with SenC, Csp3 with Cyto_bo, PcuAC and Cu-oxidase_4, and Csp1/2 with DUF386. Some of those may provide indications on the putative functions of little characterized copper-related proteins, e.g., for the assembly of specific complexes.

### Classification of β Proteobacteria According to Their Copper-Related Profiles

**FIGURE 5:**
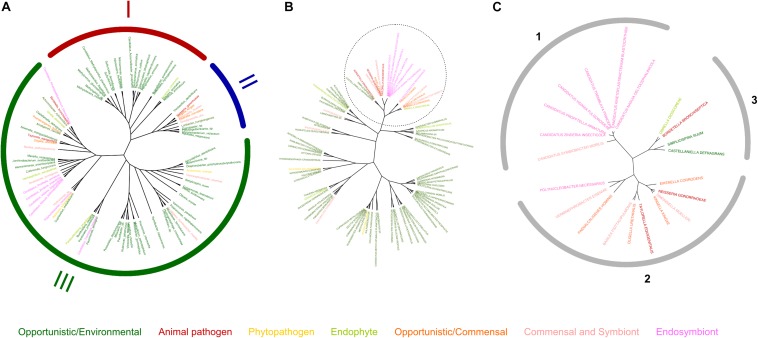
Hierarchical clustering of β proteobacteria based on their copper-related proteomes. **(A)** Phylogenetic tree based on the 16S RNA sequences. For readability, the branches of the tree do not represent true phylogenetic distances, and only one species of *Burkholderia* (*B. cepacia*), *Bordetella* (*B. bronchiseptica*), and *Neisseria* (*N. gonorrhoeae*) were used in these analyses. The tree thus comprises 86 species that form three large phylogenetic groups, namely Rhodocyclales and Nitrosomonadales (I), Nesseriales (II), and Burkholderiales (III). **(B)** Tree representation of the hierarchical clustering of the 86 bacterial species based on their sets of copper-homeostasis proteins (composition and numbers). The color code is as follows: dark green, environmental and opportunistic/environmental bacteria; pale green, endophytes; yellow, phytopathogens; salmon pink, commensal bacteria and symbionts; pink, endosymbionts; orange, opportunistic/commensal bacteria; red, animal pathogens. **(C)** The upper branch of the tree shown in **(B)** (21 species) was further analyzed by hierarchical clustering based on the complete sets of copper-related proteins in the bacteria present in that branch. The results are represented as a tree. The three groups that result from this analysis are (1) bacteria fully dependent on host cells (endosymbionts); (2) extracellular bacteria that depend on hosts; and (3), environmental bacteria. See text for details.

We also performed clustering of the bacterial species based on their respective copper-related proteomes and compared this classification with a 16S-RNA-based phylogenetic tree (Figures 5A,B). This analysis was performed on 86 species as above to avoid overrepresentation of the *Burkholderia*, *Neisseria* and *Bordetella* genera. As our results have indicated that the copper homeostasis protein complements found in β proteobacteria appear to correlate with lifestyles better than cuproproteins or chaperones, we first used the homeostasis subset of copper-related proteins to perform hierarchical clustering. Interestingly, those analyses yielded a tree in which bacteria with a host-associated lifestyle form a separate cluster (top branch in Figure 5B) from environmental bacteria, which form several other large clusters, most likely related to their niches. Outliers include *O. formigenes*, a commensal with a very different set of copper-related proteins than other commensals, and two other bacteria. Hierarchical clustering gives rather different results than the 16S-RNA-based phylogenetic tree with the same 86 species (Figure 5A).

In an attempt to refine the sorting of bacteria with host-associated lifestyles, we performed a second round of hierarchical clustering with the subset of bacteria (21 species) found in the upper branch of the tree shown in Figure 5B, this time based on their entire complements of copper-related proteins. This analysis revealed distinct groups (Figure 5C). Those found in group 1 are totally dependent on a host cell. They are all endosymbionts, except for *Candidatus S. mobilis*, which forms a consortium by maintaining cell-to-cell contact with *Chlorobium chlorochromatii*, a non-motile photolithoautotrophic green sulfur bacterium (see Supplementary Table S1). Unlike *C. chlorochromatii*, *Candidatus S. mobilis* fully depends on this symbiosis. Bacteria found in group 2 are extracellular but host-dependent bacteria, i.e., symbionts, commensals, and pathogens. One exception is *Polynucleobacter necessarius*, an endosymbiont of a protist. Its large proteome and its free living *Polynucleobacter asymbioticus* relative suggest that this symbiosis evolved recently. Bacteria found in group 3 live in the environment, even if one of them, *B. bronchiseptica*, is also an animal pathogen. Altogether, thus, the known copper-related proteomes of β proteobacteria correlate reasonably well with their lifestyles and niches.

#### Copper-Related Proteins in Specific β Proteobacterial Genera

We took advantage of the availability of the full genomic sequences of large numbers of species of *Bordetella* (15), *Burkholderia* (11), and *Neisseria* (10) to perform more detailed analyses of the relationship between lifestyle and copper-related proteomes. All *Neisseriae* have small-size proteomes and are mostly commensals (Liu et al., 2015). However, *N. meningitidis*, a commensal of the human nasopharynx, is responsible for life-threatening meningitis or sepsis when it breaches the epithelial barrier, and *N. gonorrhoeae*, an obligate human-restricted pathogen, infects the genital tract. In contrast, the 11 species of *Burkholderia* all have large proteomes. They are environmental species, phytopathogens, or patho-opportunists that can cause serious infections (Mahenthiralingam et al., 2005; Cui et al., 2016). With 15 representatives, the genus *Bordetella* displays more varied lifestyles, including obligate host-restricted pathogens, environmental species, wide-host-range pathogens, commensals, opportunists, and uncharacterized species (Linz et al., 2019) (Supplementary Table S1).

**FIGURE 6:**
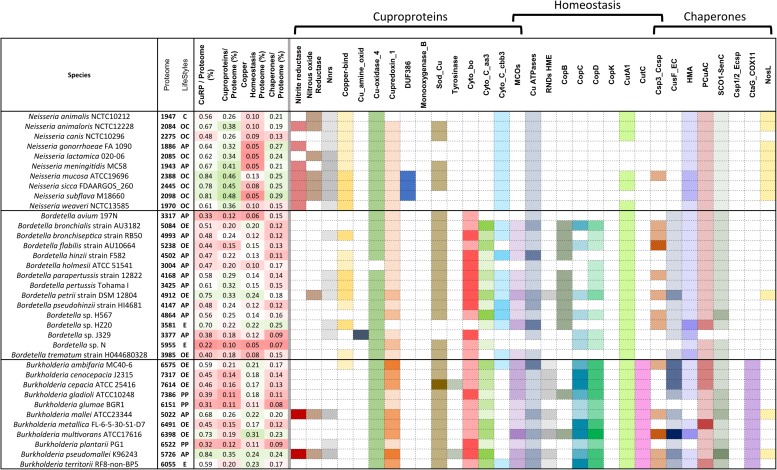
Copper-related proteomes of various *Bordetella*, *Burkholderia*, and *Neisseria* species. See the legend to Figure 2 for details.

The proportions of copper-related proteins relative to the total proteomes vary more widely among *Burkholderiae* and *Bordetellae* than among *Neisseriae* (Figure 6). Environmental and opportunistic species of the three genera generally have more cuproenzymes, in particular involved in aerobic respiration in most *Bordetella* and *Burkholderia* species, than obligate pathogens or commensals. In contrast, *Neisseriae* have few Cu-containing subunits of aerobic respiratory chains, but they harbor other cuproenzymes, i.e., nitrite reductase and/or N_2_OR, that are absent from most *Bordetellae* and *Burkholderiae*. *Neisseriae* are devoid of MCOs, unlike the other two genera. Interestingly, plastocyanin-like proteins are found in all *Neisseriae* and a majority of *Bordetellae*, but they are absent from all but one *Burkholderia*.

Major differences between lifestyles are again reflected in the sets of copper homeostasis proteins. *Neisseriae* have shed most of their Cu homeostasis systems, which represent only between 7 and 18% of their copper-related proteomes, much lower than the β–proteobacterial median (27%). This contrasts starkly with *Burkholderiae*, that are replete with copper homeostasis proteins and chaperones. In that group, *Burkholderia multivorans* has the largest proportions of total copper-related proteins and of copper homeostasis systems, while phytopathogens, *Burkholderia glumae* and *Burkholderia plantarii*, have fewer such proteins.

*Bordetellae* present more varied patterns of copper-related proteins than the other two genera. Obligate, host-restricted pathogens *Bordetella holmesii* and *Bordetella avium* have the smallest proteomes and very few defense systems, in contrast with environmental *Bordetella petrii*, which has the largest proportions of cuproproteins, copper homeostasis systems, and copper chaperones. Interestingly, differences are conspicuous among environmental species in their complements and copy numbers of copper-homeostasis proteins, suggesting specific adaptations to distinct niches by horizontal gene transfers or gene duplications. For instance, *Bordetella flabilis* has one of the largest proteomes among environmental *Bordetellae* but fewer copper-homeostasis systems than *B. petrii* or *Bordetella bronchialis*, possibly indicating that it is in the process of adaption to a more restricted niche. *Bordetella* sp. N has the largest proteome of *Bordetellae* so far, which predicts an environmental niche. However, it has hardly any homeostasis systems or chaperones, at odds with a *bona fide* environmental lifestyle. Another intriguing isolate is *Bordetella* sp. J329 isolated from a patient. It is devoid of Cyt C oxidases and SodC, which are rare features among *Bordetellae*.

### Copper-Related Proteomes in Other Proteobacteria

**FIGURE 7:**
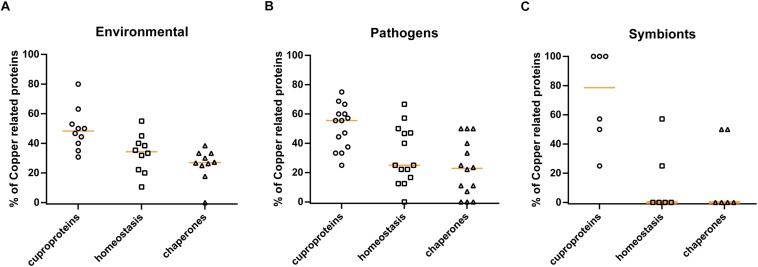
Profiles of copper-related proteomes as a function of the lifestyles of other proteobacteria. The predicted copper-related proteomes of a sample of α and γ proteobacteria were analyzed. The 30 species used in this analysis and their full sets of data are shown in Supplementary Table S3. Three groups (panel **A**: environmental species, panel **B**: pathogens, and panel **C**: symbionts) were made according to lifestyles, and the proportions (in%) of cuproproteins, copper-homeostasis proteins, and copper chaperones relative to the complete copper-related proteomes were determined for each species. The orange horizontal lines represent the medians of the values for the various groups.

Finally, to determine whether our findings with β Proteobacteria could be generalized to other phylogenetic groups, we selected 30 species of α and γ Proteobacteria of various lifestyles (pathogens, symbionts, and environmental species), with fully sequenced and assembled genomes, and we analyzed their copper-related proteomes as above (Supplementary Table S3). Similar to β Proteobacteria, environmental species have larger proportions of their copper-related proteomes dedicated to homeostasis than the other groups, and symbionts have hardly any copper homeostasis systems and chaperones (Figure 7). However, differences between pathogens and environmental species are less marked in this analysis, most likely because of the limited sample size and because several species defined as pathogens are also environmental, such as *Legionella pneumophila* and *Vibrio cholerae*. Altogether, thus, the trends observed in this smaller set confirm the correlation between lifestyles and copper homeostasis.

## Discussion

In the evolution of life on Earth, the use of copper has been linked to the appearance of molecular oxygen, as oxidation of insoluble Cu(I) to soluble Cu(II) made copper bio-available for enzymatic oxido-reductions, hydrolysis reactions, and electron transfer chains (Solioz, 2018). Most aerobic or facultative aerobic bacteria use copper, as several protein complexes involved in aerobic respiration, i.e., cytochrome bo ubiquinol oxidases, type cbb3 cytochrome C oxidases and type aa3 cytochrome C oxidases, include copper-containing subunits. In contrast, many anaerobes do not use copper at all and thus in general, aerobic bacteria have larger cuproproteomes than anaerobic ones (Ridge et al., 2008). The link between oxygen and copper utilization is a reason why the overwhelming majority of β proteobacteria have cuproproteins, with the exception of endosymbionts that have shed most of their metabolic capacities. Indeed, most β proteobacteria in our set are aerobes or facultative aerobes. Median numbers show that the proportions of cuproproteins relative to the sizes of the predicted proteomes are rather similar between pathogens, commensals, and environmental bacteria, but smaller in symbionts and endosymbionts. This is also broadly true for copper chaperones, many of which are involved in assembly of energy-generating complexes.

In β Proteobacteria, two factors appear to determine the proportions of proteins involved in copper homeostasis: the size of the global proteome of each species and its lifestyle. Unsurprisingly, bacteria with larger proteomes generally have more defense systems than smaller-proteome species, as they probably live in less constant environments and thus need to adapt to a variety of stressful conditions, possibly including high concentrations of toxic metals. The sizes of copper-homeostasis proteomes are mainly determined by the lifestyles of the species. Thus, as a general rule, environmental bacteria have relatively large proportions of their proteomes dedicated to copper homeostasis, irrespective of the sizes of their total proteomes. Copper-homeostasis proteins are more abundant relative to the total proteomes, by a factor of three, in environmental bacteria than in the other bacterial groups. The evolution from environmental bacteria to pathogens, commensals, and symbionts in β proteobacteria is globally characterized by the shedding of copper homeostasis systems. In other words, natural selection specifically favors the elimination of copper defense genes from bacteria in the course of their adaptation to eukaryotic hosts niches. This appears to be the case in other Proteobacteria as well.

However, absence of known copper homeostasis genes does not preclude the existence of other, yet unidentified homeostasis systems. Non-specific systems might also be involved, as exemplified by yersiniabactin in *E. coli*, a siderophore that also participates in copper homeostasis (Chaturvedi et al., 2012; Koh et al., 2017). Other small molecules such as glutathione, bacillithiol, and mycothiol may also play important roles in metal tolerance (Helbig et al., 2008).

Among β Proteobacteria, two groups of bacteria harbor greater than average numbers of copper-homeostasis genes: those that live in environments polluted by metals, and Nitrosomonadales. The first group is exemplified by extremely resistant organisms such as *C. metallidurans* or *H. arsenicoxidans*, which thrive in soils heavily contaminated with transition metals thanks to large numbers of export, storage, and detoxification systems. Overrepresentation of copper-related proteins involved in homeostasis relative to cuproproteins and copper chaperones in environmental bacteria corroborates the idea that the use of copper as a cofactor and the defense against copper are not correlated but evolved independently, as in other phylogenetic groups (Solioz, 2018). Note that *C. metallidurans* and *H. arsenicoxydans* also have larger proportions of cuproproteins than most β proteobacteria, in part because they have four or five MCOs, cuproproteins that also contribute to protection against copper.

Another factor that is likely to strongly contribute to the abundance of copper defense systems in environmental bacteria is the necessity to survive predation by protozoa in soils and water. The relationship between protozoa and bacteria is a long-standing one, and copper is part of the arsenal used by the former to poison and kill their bacterial preys (Hao et al., 2016). In consequence, there is a strong correlation between the presence of copper efflux systems in bacteria and their ability to survive in amoeba and other protozoa. Those systems in turn may contribute to the ability of environmental bacteria to cause opportunistic infections in specific settings such as in immunocompromised hosts. Indeed phagocytes, which use transition metals to kill invading microbes as part of the innate immune response (Fu et al., 2014; Djoko et al., 2015), share pathways of intracellular copper trafficking with protozoa (Hao et al., 2016). It is thus very likely that the abundance of copper homeostasis systems in many environmental β proteobacteria has been selected for by the need to resist killing by protozoa, and those defenses then enabled opportunists to resist killing by phagocytic cells in their occasional mammalian hosts.

Pathogenic bacteria of mammals represent a special case with respect to copper. Copper is sequestrated away from the pathogens by host proteins in mucosa or body fluids, and therefore bacteria need to specifically acquire it for assembly of their own cuproproteins. On the other hand, as outlined above, the innate immune system uses copper as a line of defense, with macrophages importing copper into the phagolysosome compartment as a means to destroy bacteria, together with oxidative and nitrosative stress and antimicrobial peptides. Animal pathogens must thus strike a fine balance to deal with copper starvation or excess depending on the specific environments encountered in their hosts. We have started to address this issue with the host-restricted, obligate pathogen *B. pertussis* and discovered that it has lost a number of genes coding for defense systems present in model bacterial pathogens. According to phylogenetic classifications, *Bordetellae* are close to an environmental/opportunist species, *Achromobacter xylosoxidans*, and to an endosymbiont, *Candidatus Kinetoplastibacterium blastocrithidii*. Comparisons between *A. xylosoxidans* (genome: 7.3 Mb), *K. blastocrithidii* (0.86 Mb), *B. pertussis* (4.086 Mb), and *B. bronchiseptica* (5.34 Mb), the latter of which can both infect mammals and survive in the environment, show that *A. xylosoxidans* has considerably more Cu-ATPases, HME transporters, MCO and other homeostasis systems than the two *Bordetella* species, but only slightly more cuproproteins and chaperones. *B. bronchiseptica* is in an intermediate situation regarding its defenses against copper (see below). Unlike *B. pertussis*, *B. bronchiseptica* has two distinct but interconnected cycles, in mammalian hosts and in amoeba (Taylor-Mulneix et al., 2017). Protozoan predation may thus have positively selected for the copper homeostasis systems that remain in *B. bronchiseptica* but are no longer functional in *B. pertussis*. At the other end of this spectrum, the endosymbiont *K. blastocrithidii* has no homeostasis systems. Such a scenario fully supports the global trends observed on our large sample of β proteobacteria.

Interestingly, however, most differences between *B. pertussis* and *B. bronchiseptica* with respect to copper-related systems are not found in their genomes but at the level of transcription (our unpublished observations). Thus, *B. pertussis* possesses genes coding for a Cu-ATPAse and an MCO that are no longer expressed and regulated by copper, while in *B. bronchiseptica* both are strongly upregulated. *B. pertussis* has thought to have derived by genomic reduction from a common ancestor close to current-day *B. bronchiseptica* (Diavatopoulos et al., 2005), and our observations support the idea that streamlining of the *B. pertussis* genome is an on-going process. Thus, a caveat of *in silico* genomic analyses is that they can only reveal which genes are present or absent, but not whether they are functional.

With very small total proteomes and few copper-related proteins, endosymbionts represent one extreme of our bacterial set. The proportions of cuproproteins relative to their total proteomes are hardly lower than in other β proteobacterial groups, and thus those genes appear to follow the general genomic reduction toward a symbiotic lifestyle. The proportions of their copper-homeostasis proteins relative to their total proteomes are markedly lower than in other bacterial groups, indicating that those genes are shed faster than others. Genes coding for chaperones tend to follow the same route. If chaperones were only providing copper for cuproenzyme assembly, one would expect them to decrease in similar proportions as genes for cuproproteins. That they are lost in greater proportions suggests that some of them also served defense purposes, likely by sequestration, in the bacterial ancestors of those symbionts.

Copper is increasingly used as an antibacterial agent, notably in hospitals, in agriculture, and in animal breeding (Grass et al., 2011; Lemire et al., 2013; Schmidt et al., 2016). As shown here, environmental bacteria are well-equipped to deal with such aggressions. Alarmingly, such bacteria cause a growing number of opportunistic infections, including *C. metallidurans* and other β proteobacteria that we have classified as “environmental opportunists” based on our review of the literature (Langevin et al., 2011; Bayhan et al., 2013; Bilgin et al., 2015; Jardine et al., 2017). Finding ways to limit the emergence of new opportunists favored by widespread use of copper as an antibacterial agent will thus become increasingly important. Finally, our study includes a number of bacterial species with bioremediation potential. Deciphering the interplay between metallic stress and stress caused by toxic chemicals might help make the best use of such organisms.

## Conclusion

*In silico* analyses of the predicted copper-related proteomes of a large panel of β proteobacteria have indicated that lifestyle shapes the copper-related proteome, and this is particularly reflected in systems involved in copper homeostasis. Evolution from environmental niches toward commensalism, obligate pathogenesis or symbiosis parallels the loss of copper-homeostasis systems. Endosymbionts represent an extreme situation with respect to copper, having lost almost all copper-related genes with the exception of few cuproproteins involved in electron transfer. The correlation between lifestyle and copper homeostasis appears to hold true in other groups of Proteobacteria as well.

## Data Availability Statement

All datasets generated for this study are included in the manuscript/Supplementary Files.

## Author Contributions

RA and FJ-D conceived the study. RA, AR-M, and GR gathered the data and prepared the figures and tables. FJ-D wrote the manuscript. All authors analyzed the data and reviewed the manuscript.

## Conflict of Interest

The authors declare that the research was conducted in the absence of any commercial or financial relationships that could be construed as a potential conflict of interest.

## Footnotes

1 hmmer.org

2 http://www.tcdb.org/download.php

3 https://www.jcvi.org/tigrfams

4 http://signalfind.org/tatfind.html

5 http://bonsai.hgc.jp/~mdehoon/software/cluster/software.htm

6 tree.bio.ed.ac.uk/software/figtree/

7 eddylab.org/infernal/

8 https://www.megasoftware.net

## Supplementary Material

FIGURE S1 Full complements of copper-related proteins (cuproproteins, copper-homeostasis proteins, and copper chaperones) in the entire set of β proteobacteria analyzed in this study. The sizes of the proteomes (numbers of proteins) and the lifestyles are given in the second vertical panel. E, OE, AP, PP, OC, C, S, EnP, and EnS represent environmental, opportunistic/environmental, animal pathogen, phytopathogen, opportunistic/commensal, commensal, symbiotic, endophytic, and endosymbiotic bacteria, respectively. In the third vertical panel are shown the proportions (in %) of the copper-related proteomes (CuRP) relative to the full proteomes for each species, and the proportions (in %) of the proteins in the three functional categories (cuproproteins, copper-homeostasis proteins, and copper chaperones) relative to the complete proteomes. In that panel, the white, green, and red background colors represent average values, above-average values and below-average values, respectively, relative to the 119 species, with the intensities of the colors relating to the distances to the average values. The abundances of each type of proteins in each bacterial species are represented in the last panel. Note that MCOs are both cuproproteins and copper- homeostasis proteins, and Csp3s (cytoplasmic copper storage proteins) are both copper-homeostasis proteins and copper chaperones. The intensities of the colors increase with the numbers of paralogs in each bacterial species, with white indicating the absence of the protein. The absolute numbers of proteins of each type in each bacterial species can be found in Supplementary Table S2.

TABLE S1 β proteobacterial species analyzed in this study. The species are sorted according to phylogenetic analyses, and the references used to determine their lifestyles are indicated along with the links to the corresponding publications. The different lifestyles are represented by colors: dark green for environmental species (E), green for opportunist/environmental species (OE), red for animal pathogens (AP), yellow for phytopathogens (PP), light beige for commensal species (C), orange for opportunist/commensal species (OC), beige for symbionts (S), light green for endophytes (EnP) and pink for endosymbionts (EnS). The sizes of the predicted proteomes are also provided.

TABLE S2 Copper-related proteomes of β proteobacteria. The list of the 119 species analyzed in this study is shown. The species are sorted according to phylogenetic analyses. The GenBank accession numbers of the replicons are given as well as their total sizes in megabases (Mb). The GC content (% GC) and the sizes of the proteomes (numbers of predicted proteins) are also reported. For each Pfam domain, the number of occurrences in each species is given as well as the locus_tag name (ID xxxx) found in the corresponding GenBank file. For respiratory components no locus_tag is given but only the number of occurrences of these multi-component systems.

TABLE S3 Copper-related proteomes of α and γ proteobacteria. The list of the 30 species analyzed in this study is shown. The GenBank accession numbers of the replicons are given as well as their total sizes in megabases (Mb). The GC content (% GC) and the sizes of the proteomes (numbers of predicted proteins) are also reported. For each Pfam domain, the number of occurrences in each species is given as well as the locus_tag name (ID xxxx) found in the corresponding GenBank file. For respiratory components no locus_tag is given but only the number of occurrences of these multi-component systems.

FILE S1 HMM profile for the copper storage protein domain, CSP.hmm.
