# Supplementary figures and images for: Relationships Between Copper-Related Proteomes and Lifestyles in β Proteobacteria
Source: Front Microbiol. 2019 Sep 24;10:2217. doi: 10.3389/fmicb.2019.02217 (PMC6769254; doi:10.3389/fmicb.2019.02217)

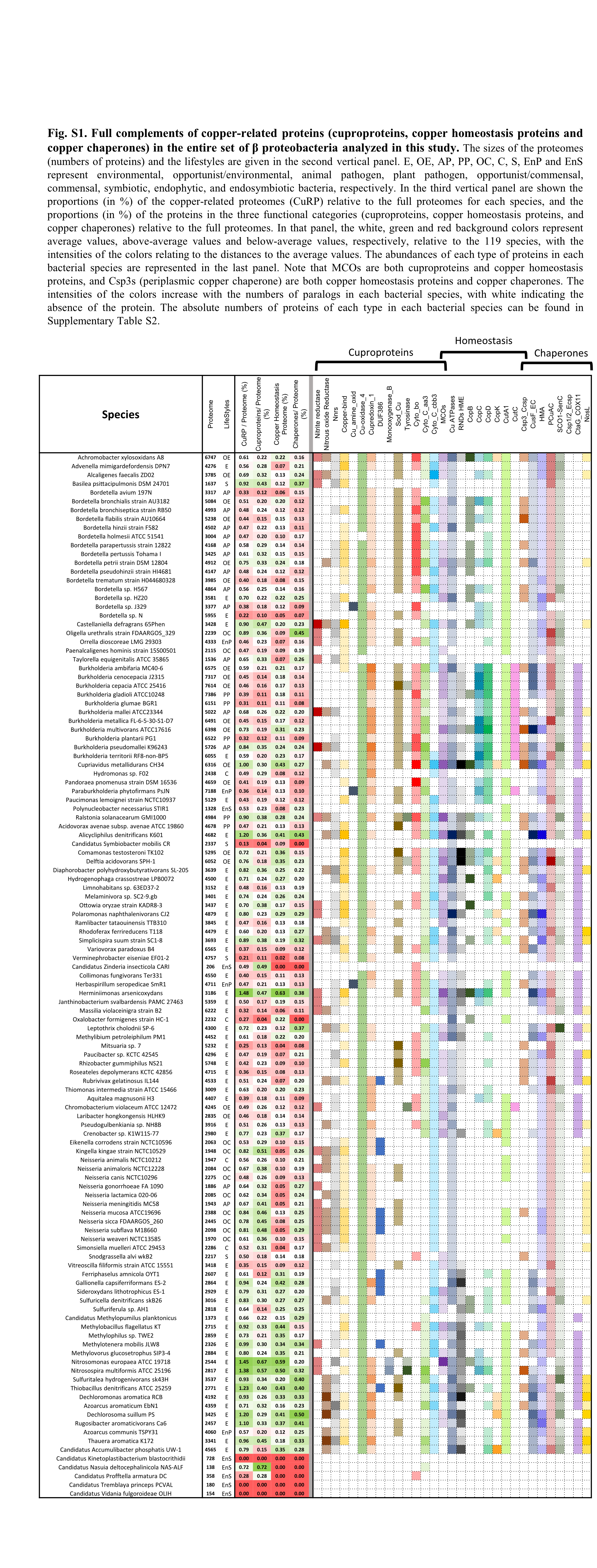

Supplement: FIGURE S1 — Full complements of copper-related proteins (cuproproteins, copper-homeostasis proteins, and copper chaperones) in the entire set of β proteobacteria analyzed in this study. The sizes of the proteomes (numbers of proteins) and the lifestyles are given in the second vertical panel. E, OE, AP, PP, OC, C, S, EnP, and EnS represent environmental, opportunistic/environmental, animal pathogen, phytopathogen, opportunistic/commensal, commensal, symbiotic, endophytic, and endosymbiotic bacteria, respectively. In the third vertical panel are shown the proportions (in %) of the copper-related proteomes (CuRP) relative to the full proteomes for each species, and the proportions (in %) of the proteins in the three functional categories (cuproproteins, copper-homeostasis proteins, and copper chaperones) relative to the complete proteomes. In that panel, the white, green, and red background colors represent average values, above-average values and below-average values, respectively, relative to the 119 species, with the intensities of the colors relating to the distances to the average values. The abundances of each type of proteins in each bacterial species are represented in the last panel. Note that MCOs are both cuproproteins and copper- homeostasis proteins, and Csp3s (cytoplasmic copper storage proteins) are both copper-homeostasis proteins and copper chaperones. The intensities of the colors increase with the numbers of paralogs in each bacterial species, with white indicating the absence of the protein. The absolute numbers of proteins of each type in each bacterial species can be found in Supplementary Table S2. [file Image_1.tiff]
